# The Patients' Christmas Week

**Published:** 1912-01-06

**Authors:** 


### THE PATIENTS' CHRISTMAS WEEK.

We publish this week a further instalment of Christmas Festivities in hospitals, and it will be seen from these additional descriptions that several hospitals have taken to heart our advice on the importance of using these Christmas entertainments as a means for securing further useful publicity to the institutions concerned. The lay papers show that accounts of Christmas in the hospitals are often perfunctory; they should be used to draw attention in the most practical way not merely to the enormous amount of work done voluntarily on these occasions, but to the voluntary workers themselves, the body of whom do so much that is unappreciated by the general public. Once a precedent is created for bringing home to the world outside the services of the general rank and file of hospital staffs on these occasions, public support will be strengthened, and Christmas is a time for strengthening that support in a way that is likely to attract the admiration of many who are deaf to appeals in their general form. Such grounds as these have led us to throw open our columns at whatever reasonable length the hospitals have been ready to make use of them. It will be seen that the response this year to this new method of appeal promises well for the future. We believe that on this question The Hospital can do, as our two issues have shown, what is found impossible in the crowded columns of the lay press, to obscure corners of which, as a rule, the accounts of Christmas Festivities are relegated. By the time next Christmas comes round we hope that the lesson may be fully learnt, and that the pioneer hospitals which have seized their opportunity on this occasion will be followed by the general mass of institutions, who will thus benefit, as the more enterprising have done already, by the new opportunity we have thrown open to them of exploiting a new appeal to public support.

### Waxworks at West Ham.

Christmas began at the West Ham and Eastern General Hospital, Stratford, on Saturday, the 23rd ult., with a reception of benefactors and friends to witness the distribution of presents from the Christmas tree to the patients. The matron, Miss Sordy, and the Secretary, Mr. A. W. Scrivener, received in the Entrance Hall. The distribution from the Christmas tree took place in the Children's ward, where a splendid Christmas tree laden with good things was in evidence. "Father" Christmas and "Mrs." Christmas, Dr. Ledger and Dr. McIntyre respectively, were introduced by the Chairman, Mr. T. A. Cook, and proceeded, assisted by the staff, to give presents to all the patients. The children's presents consisted of toys; the men's and women's of articles of clothing, many of these being provided by the Duchess of Marlborough, the Countess of Warwick, and Mr. Alfred de Rothschild. It would be invidious to say which ward looked the best, as equal care had been taken by all the sisters, but naturally the newer wards lent themselves most easily to decorative effects; and nothing more fairy-like could be imagined than the Marlborough and the Annie Zunz wards—the two most recently added to the hospital. Among the guests present were the Bishop of Barking; the Rev. F. J. Key, Honorary Chaplain to the Hospital, and Mrs. Key; Mr. T. A. Cook, Chairman, and Mrs. Cook; the Hon. Treasurer, O. B. Doherty, Esq.; Mrs. Ellis Fredericks, and a host of others.

It has been usual for the nurses to make a collection to defray the expenses; but on this occasion they preferred, by hard work, to prepare useful articles for sale to the guests, and there was a stall in the Entrance Hall, presided over by the out-patients sister, at which useful presents (a great many of them made by the nurses themselves) were for sale. A flower stall just outside the Annie Zunz ward, a bran tub in another ward, and a cake (the weight of which might be guessed by any one who liked to contribute) were various efforts which produced a considerable sum to pay expenses of the tea and entertainment. Everybody seemed delighted with the arrangements, especially the patients; and when the men were allowed to smoke, as is customary at Christmastide, their satisfaction seemed complete. During the evening the Rev. F. J. Key, the chaplain of the hospital, addressed some cheering words to the patients, who on Monday were served with their Christmas dinner. Dr. Nicoll, Dr. Thompson, Dr. Challans, and Dr. Couzens carved the turkeys in the various wards, and assisted in the distribution of the dinner.

In the evening Marlborough ward entertained all the patients who were able to be moved to a tea and entertainment. The entertainment consisted of "Mrs. Jarley's Waxworks." Mrs. Jarley (Dr. Windsor) was a wonderful characterisation, speaking in a broad Lancashire dialect and keeping the patter going without hesitation. "She" was assisted by Jarge and Jean—a house surgeon and sister respectively—who worked without a pause, and whose gag and business were never deficient in quality.

The same entertainment was repeated on Tuesday evening in the Zunz ward, and it was interesting to hear the various sisters come and ask if they might bring some more of their patients down from their wards. The doctors gave permission as liberally as they could, and the ward was crammed with patients who had been interested the night before, or who had heard the fame of the entertainment, and insisted on coming, where they could be allowed. Sunday was kept as a quiet day, the Chaplain attending at the usual service in the afternoon; and the patients' friends coming in to visit and see the decorations.

### The Crib at Cambridge.

Our celebrations began on Thursday, December 21, when the choir of Emanuel Congregational Church sang carols in the wards of Addenbrooke's. On Friday carols were again sung in all the wards by the choir of the University Church, St. Mary's the Great, and the nurses. The members of the choir marched in procession, each carrying a Japanese lantern alight on canes about four feet high. This is a distinct feature of Christmas here, and is greatly appreciated and admired. On Saturday and Sunday the chaplain held services in most of the wards.

In Bowlen ward (male accident) the brackets of the bed pulls over each bed were decorated with ivy, and the pulls were generally covered with gilt-paper. The window sills were gaily adorned with choice coloured fairy-lamps, and the hanging electric lamps were covered with pink silk shades edged with silver tinsel, giving a pleasing effect. These shades are the handiwork of the ward sister, Miss Paterson, and her band of enthusiastic nurses. The arch of the central fireplace in this ward was illuminated with a fancy electric lamp, and the base was tastefully decorated with choice flowers and plants, with a further display of very prettily-shaded fairy-lamps. In Griffith ward (male medical) the brackets of the bed pulls were prettily decorated with ivy and the ropes covered with richly-shaded material. The window sills here again were decked out with plants, flowers, ferns, and fairy-lamps. The hanging electric lamps were adorned with white crimped paper shades; these were again decked with red poppies,, all the handiwork of a most enthusiastic ward sister, Miss Anderson, and her staff.

In Albert ward, with its twenty-nine male surgical beds, the decorations were similar yet distinctive, for this ward differed from the others in that it had a crib illustrating in model the Nativity. This was for a second time lent by a friend of the hospital. On each window-sill there was a fern in the midst of a cluster of fairy-lamps. The bed-pulls and brackets were decorated, and from each bracket was suspended a Japanese lantern. The ward tables were furnished with candles, fairy-lamps, and flowers. The six white columns over the central fireplace were entwined with ivy, and the base decked with flowers, plants, and fairy-lamps. The hanging electric lamps were covered with combinations of snow-white and scarlet paper shades, made by the ward sister, Miss Wethered.

In the women's surgical Victoria ward there was a choice selection of flowers. The bed-pull brackets and the ropes of the bed-pulls were decorated with ivy. From each bracket hung a very large Japanese lantern. The window-sills were decorated with plants, fairy-lamps, etc. The hanging electric lamps were covered with pink paper shades, the handiwork of the enthusiastic sister, Miss Laughton, and her helpers. The ward tables were gaily decorated with fairy-lamps, candles, and flowers. The Christmas tree was in this ward, and stood thirteen feet, being illumined by means of electric light, by the kindness of the local Electric Supply Company. In Hatton women's medical ward the decorations were much the same as those described, except that the hanging electric lamps were covered with pink silk shades, each having suspended from the edges clusters of crystalline beads.

On Christmas Day presents were distributed in the morning by Father Christmas. At twelve o'clock dinner was served in all the wards, consisting of roast turkey, plum pudding, fruit, sweets, and Christmas crackers, each ward having its own special carver: Bowtell ward, Dr. H. Gibson, second house surgeon; Griffith ward, Dr. Alec Gill, house physician; Albert ward, Michael Coles, the Secretary-Superintendent; Victoria ward, Dr. Dummere, house surgeon; Hatton ward, Dr. Searle, general practitioner and son of the late Master of Pembroke College. After dinner the men were allowed to smoke at ease, and during the afternoon a nigger troupe went round the ward singing nursery rhymes. The troupe was composed of our own nurses entirely, and their performances caused the greatest fun. In all the wards there were special teas, accompanied with gramophone music, with the exception of Victoria ward, where the Christmas tree was. After tea came Father Christmas the Rev. C. Grant-Cowan, headed by a drum and trumpet band. His long and tedious duty, lasting nearly two hours, was carried out in an admirable manner and to the great delight of all.

The porters had their Christmas dinner with the patients in Albert ward. The maids had their Christmas dinner of turkey and plum pudding and dessert served to them at choicely decorated tables in the nurses' dining-hall. On Boxing Day there was a special tea and concert in Griffith ward, the concert being given by Mr. and Mrs. Alban Young, Mr. Paul MacAlister, F.R.I.B.A., and other friends. As many patients as possible were brought from other wards.

### An East Anglian Christmas.

The Yuletide and New Year festivities at the East Suffolk and Ipswich Hospital were of an exceptionally pronounced character, and the response to the matron's appeal for funds and gifts in kind was both prompt and generous. Although the work of this hospital has been somewhat congested lately owing to an unusual influx of accidents and in-patients generally, and a consequently long waiting list, the fact that its resources were somewhat taxed did not mar the enjoyment of the inmates. The Christmas fare in the wards was varied and liberal, while the amusements left little to be desired. An excellent concert was given on Thursday, December 28, by an orchestra of local ladies under the baton of Mr. W. Hockey, F.R.C.S. Dr. Sydney Williams, the senior house surgeon, was undoubtedly the star of the vocalists, especially if the vociferous plaudits of his patients were to be taken as a criterion. It has been the custom of this hospital to have a Christmas-tree a few days after Christmas Day, and this enables many of the hospital's best supporters to be present who otherwise would not attend. The tree, which was of somewhat gigantic proportions, was given, as usual, by Mr. E. G. Pretyman, M.P., and was illuminated by the hospital's electric contractors. Among the innumerable presents with which its branches were bedecked was a Christmas box for each individual in the hospital. The Mayor and Mayoress of Ipswich, Mr. and Mrs. F. E. Rands, distributed the presents, assisted by the Chairman of the Board, Mr. F. Messent; the Secretary, Mr. A. Griffiths; and members of the staff. Each ward was decorated in a different way, thanks to the artistic talents of the sisters in charge, while the general arrangements made by the matron were excellent. Saturday, December 30, was given over to the entertainment of the children, of whom there were some dozen more in the wards than usual. The programme included a special tea-party, games, music, and a magic-lantern. The festivities concluded on New Year's Day with a domestic staff-tea.

### At Beckett Hospital, Barnsley.

Festivities at Beckett Hospital, Barnsley, this year have been of a very elaborate nature. Owing to extensive alterations one of the large wards is at present unoccupied, and this was devoted entirely to entertaining the convalescent patients. A nice and commodious temporary stage was erected at one end, which has been occupied very often during the Christmas holidays by local talent for the benefit of the patients and their friends. The matron, Miss Edmondson, who is the life of the institution, along with the sisters and nurses, has spared nothing in making everyone as bright as possible during the past week. On Boxing Day the Mayor and Mayoress attended the usual Christmas-tree gathering and distributed gifts of clothing to every patient in the institution, which has many friends who never forget to send kind gifts to the hospital, the consequence being that old and young are laden with much appreciated gifts. During the week choralists, glee parties (a well-known local feature), and others have given their services, and on Friday night a moving-picture entertainment was given by the Barnsley Princess Palace Company. On Tuesday last the nursing staff occupied the stage and rendered in a very pleasing manner a programme of songs, a one-act farce, called "Clothes Mangled Here," and an ambitious operetta by J. L. Roeckel, entitled "The Hours," the whole being produced under the supervising care and tuition of the matron, Miss Edmondson.

### A Precedent Created.

The Duke of Devonshire on Christmas Day sent £50 as "Mayor of Chesterfield" to the Chesterfield and North Derbyshire Hospital. This, we understand, establishes a precedent which, it is hoped, in future may be followed.

